# Metabolomic Tools for Secondary Metabolite Discovery from Marine Microbial Symbionts

**DOI:** 10.3390/md12063416

**Published:** 2014-06-05

**Authors:** Lynsey Macintyre, Tong Zhang, Christina Viegelmann, Ignacio Juarez Martinez, Cheng Cheng, Catherine Dowdells, Usama Ramadan Abdelmohsen, Christine Gernert, Ute Hentschel, RuAngelie Edrada-Ebel

**Affiliations:** 1 Strathclyde Institute of Pharmacy and Biomedical Sciences, University of Strathclyde, 161 Cathedral Street, Glasgow G4 0RE, UK; E-Mails: tong.zhang.101@strath.ac.uk (T.Z.); christina.viegelmann@strath.ac.uk (C.V.); gomoku13@hotmail.com (I.J.M.); c.dowdells@strath.ac.uk (C.D.); 2 Department of Botany II, Julius-von-Sachs Institute for Biological Sciences, University of Würzburg, Julius-von-Sachs-Platz 3, D-97082 Würzburg, Germany; E-Mails: cheng.cheng1@uni-wuerzburg.de (C.C.); usama.ramadan@uni-wuerzburg.de (U.R.A.); christine.gernert@mail.uni-wuerzburg.de (C.G.); ute.hentschel@uni-wuerzburg.de (U.H.)

**Keywords:** metabolomics, dereplication, symbiotic bacteria, mass spectrometry, NMR, multivariate analysis, metabolic profiling

## Abstract

Marine invertebrate-associated symbiotic bacteria produce a plethora of novel secondary metabolites which may be structurally unique with interesting pharmacological properties. Selection of strains usually relies on literature searching, genetic screening and bioactivity results, often without considering the chemical novelty and abundance of secondary metabolites being produced by the microorganism until the time-consuming bioassay-guided isolation stages. To fast track the selection process, metabolomic tools were used to aid strain selection by investigating differences in the chemical profiles of 77 bacterial extracts isolated from cold water marine invertebrates from Orkney, Scotland using liquid chromatography-high resolution mass spectrometry (LC-HRMS) and nuclear magnetic resonance (NMR) spectroscopy. Following mass spectrometric analysis and dereplication using an Excel macro developed in-house, principal component analysis (PCA) was employed to differentiate the bacterial strains based on their chemical profiles. NMR ^1^H and correlation spectroscopy (COSY) were also employed to obtain a chemical fingerprint of each bacterial strain and to confirm the presence of functional groups and spin systems. These results were then combined with taxonomic identification and bioassay screening data to identify three bacterial strains, namely *Bacillus* sp. 4117, *Rhodococcus* sp. ZS402 and *Vibrio splendidus* strain LGP32, to prioritize for scale-up based on their chemically interesting secondary metabolomes, established through dereplication and interesting bioactivities, determined from bioassay screening.

## 1. Introduction

Marine invertebrates such as sponges are a rich source of novel metabolites that are of medicinal interest due to their anti-cancer, anti-tumor, anti-viral and antibacterial properties [1,2,3,4]. However, there is a bottleneck when developing drugs from marine invertebrates. They are largely uncultivable and it is unsustainable to collect large quantities from marine habitats to facilitate the extraction of enough novel marine natural products for the supply chain, making pharmacological development difficult. Sponge-associated endosymbiotic bacteria are highly concentrated within the sponge matrix making up to 50%–60% of the dry weight of the sponge [5]. They are hypothesized to stabilize the sponge skeleton, process metabolic waste and provide chemical defense against environmental stresses such as predators and overgrowth of fouling organisms, by producing a plethora of novel secondary metabolites that may be structurally unique with interesting pharmacological properties [5,6,7], e.g., as antimicrobials [8] or anti-cancer drugs [9].

There is evidence to suggest that these microbes, which live symbiotically with the host organism, are the true source of many bioactive compounds discovered from associated marine invertebrates [5,10,11,12,13,14,15]. Some of these compounds can be produced in large quantities on a biotechnological scale using bacterial fermentation processes without the need to harvest the host organism and are therefore an economically viable and sustainable source of commercial quantities of metabolites of interest [16]. For example, the anti-tumor drug bryostatin 1, isolated from the marine bryozoan *Bugula neritina* and synthesized by the symbiotic bacterium *Candidatus Endobugula sertula* [9], is now produced using a large-scale fermentation process to ensure supply [17].

Key to the exploitation of marine bacteria as sources of novel marine natural products has been the implementation of 16S rRNA-based phylogenetic analysis which has been used extensively to provide an insight into sponge-specific microbial communities [18,19]. The development of new analytical technologies and instrumentation has made it possible to rapidly obtain a chemical fingerprint of bacterial extracts to potentially discover new natural products from only a few milligrams of material. Historically, selection of bacterial strains has relied on literature searching, genetic screening and bioactivity results [20]. However, cultivated bacterial strains from the same genus may appear morphologically identical, but may produce different, structurally diverse secondary metabolites [21,22]. In contrast, strains that appear different by morphology and 16S rRNA sequencing often produce the same secondary metabolites, making it difficult to pinpoint interesting bacterial strains before the time-consuming bioassay-guided fractionation and purification stages.

Dereplication is the rapid identification of known metabolites in a sample mixture [23,24,25]. Dereplication uses chromatographic and spectroscopic methods and database searching, for example using the MarinLit [26] and AntiBase [27] databases, to screen samples for known natural products, which saves time and reduces the possibility of redundancy during natural product discovery programs. Common dereplication methods involve using liquid chromatography coupled to a photo diode array (LC-PDA) system or LC-PDA with mass spectrometry (MS) using electrospray ionization (ESI) [28,29] or atmospheric pressure chemical ionization (APCI) as soft-ionization sources. Liquid chromatography mass spectrometry (LC-MS) high resolution instruments such as Quadrupole Time-of-Flight (QTOF) or Orbitrap provide accurate mass data (0.5%–5 ppm) with elemental composition output for a given ion [30]. This enables natural products databases to be queried in a high throughput manner, with fewer candidate metabolite IDs being observed for each feature. With a Quadrupole or an ion trap, data-dependent MS/MS and MS*^n^* can also be carried out to provide additional structural information (e.g., using a Q-TOF or LTQ-Orbitrap). TOF-based mass spectrometers enable a higher degree of certainty for identification of elemental compositions on the basis of both mass accuracy and isotope fit [28,31,32,33]. These instruments offer high sensitivity and accuracy in the ng or pg range and, on several newer-generation instruments, spectra can be obtained in positive and negative ionization modes during a single experiment.

Metabolomics is defined as the comprehensive analysis of the small molecules (MW < 1000) in a biological system under a given set of conditions [34]. At the biochemical level, the metabolome is most closely related to the phenotype, providing insight into biological function [35]. Mass spectrometry and nuclear magnetic resonance (NMR)-based metabolomics are readily applicable to natural products research, offering the ability to deal with complex mixtures in a highly efficient manner [36,37,38,39]. Metabolomics methods are combined with chemoinformatics approaches, e.g., unsupervised multivariate analysis methods, to uncover interesting variation amongst groups of samples (e.g., in terms of their *m*/*z* values for mass spectrometry data or chemical shifts for NMR data) [40]. Microbial metabolomics is readily applicable to investigate the physical state of cells [41], to investigate intracellular metabolites [40,41] and for the optimization of experimental conditions for the production of pharmacologically active compounds [23,25].

The aims of the study were to utilize metabolomics tools to investigate differences in secondary metabolite production in marine symbiotic bacteria to fast track the strain selection and dereplication processes for natural product drug discovery. LC-HRMS and principal component analysis (PCA) were used to pinpoint strains that were chemically diverse in a high throughput and untargeted manner. LC-HRMS results were then correlated with bioassay screening results to prioritize strains for drug discovery efforts. The study was designed to monitor secondary metabolite production, using extraction methodology optimized for the recovery of secondary metabolites. In comparison with other studies that compared strains from the same species [22,42], we were able to compare chemically diverse, non-related strains from four different phyla, cultured on a variety of growth media. Additionally, an Excel macro, developed in-house, was used to sort and remove features (pairs of *m*/*z* ratios and retention times) associated with the different culture media used. This reduced the difficulties in spectral interpretation that are often encountered when comparing bacterial strains grown on different culture media.

It was predicted that bacterial extracts containing the same secondary metabolites would cluster together whilst those extracts with chemically distinct metabolites would be observed as outliers using unsupervised multivariate analysis [23,39], providing a means to focus on chemically diverse extracts during dereplication. Therefore we used a combinatorial approach for strain selection, utilizing a data analysis workflow that encompassed features of dereplication and metabolomics to establish the chemical profiles of bacterial extracts in a high throughput manner. By incorporating metabolomics approaches, dereplication could be focused on chemically diverse bacterial extracts.

## 2. Results and Discussion

### 2.1. Diversity of Invertebrate-Associated Bacteria

**Figure 1:**
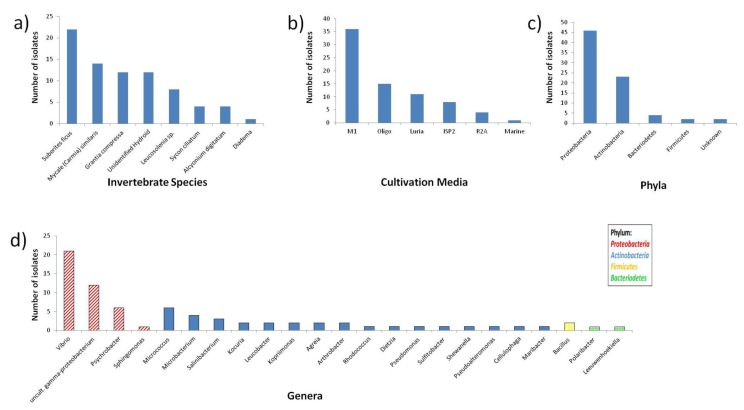
Classification of the isolates by (**a**) source invertebrate species; (**b**) cultivation media; (**c**) phyla and (**d**) by genera (if known).

Several species of cold water marine invertebrates found in Scottish coastal waters (Orkney Islands, Scotland, UK) were swabbed for microbial symbionts. Specimens were then inoculated onto various types of agar media, which yielded a total of 77 isolates (Figure 1 and Table S1 in Supplementary Information). *Suberites ficus* (sponge) yielded the highest number of isolates (22) followed by sponges *Mycale* (*Carmia*) *similaris* (14), *Grantia compressa* (12) and an unidentified hydroid (12), followed by sponges *Leucosolenia* sp. (8) and *Sycon ciliatum* (4), the soft coral *Alcyonium digitatum* (4) and sea urchin *Diadema* (1) (Figure 1a)*.* A variety of isolation media were utilized in this study to maximize the diversity of the isolates obtained. M1 obtained the highest recovery (36 isolates) whilst marine agar recovered only one isolate (Figure 1b). In terms of the diversity of isolates, M1 produced isolates belonging to 15 different genera followed by ISP2 and Luria (seven genera, respectively). Oligo (oligotrophic) media produced isolates from four genera, R2A yielded two genera and marine agar only one genus (Table S1 in Supplementary Information). This variation is consistent with the results of previous studies [43,44]. By 16S rRNA sequencing, the phylogenetic affiliations of 75 of the isolates were determined whilst a further two isolates remained unidentified (Figure 1c). The isolates were grouped to four different phyla representing 23 different identified genera (Figure 1c,d). The most abundant phylum was the *Proteobacteria* of which 42 were *Gammaproteobacteria* whilst four were *Alpha proteobacteria*, followed by the *Actinobacteria* (23), *Bacteriodetes* (4) and *Firmicutes* (2). This is consistent with the observation that it is more successful to culture *Gammaproteobacteria* than *Alphaproteobacteria* [45]. The highest numbers of isolates were affiliated to the genus *Vibrio* (21) followed by uncultured *Gammaproteobacteria* (12), *Psychrobacter* (6), *Micrococcus* (6) and *Microbacterium* (4) (Figure 1d)*.* High numbers of *Vibrio* sp. are consistent with previous studies, as they are ubiquitous in the marine environment and are associated with various algae and animals such as sponges and corals [46].

### 2.2. Data Processing and Data Clean-Up

**Figure 2:**
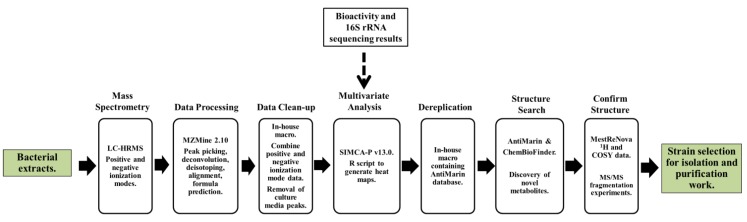
Metabolomics and dereplication workflow to aid strain selection.

Following culturing and chemical extraction, the crude extracts from the 77 bacterial isolates were subjected to metabolomic analysis according to our pre-defined metabolomics workflow pathway (Figure 2). To maximize secondary metabolite detection in this diverse bacterial population (with a range of phylogenetic affiliations and culture media), an Exactive benchtop Orbitrap mass spectrometer (Thermo Scientific, Bremen, Germany) that permitted fast polarity switching was used for untargeted dereplication. The Exactive allows positive and negative mode switching with a maximum scan time of 0.25 s and the instrument always gives good mass accuracy of <3 ppm. The average chromatographic base peak width is about 30 s; therefore, there is adequate time to acquire sufficient scans through the peak in switching mode.

Key features of the metabolomics workflow include data processing in MZmine 2.10 for peak detection, deconvolution, deisotoping, filtering (to narrow the retention time search window to 5–40 min), alignment and gap filling to make multiple data files comparable (Experimental Section 3.5 and Supplementary Information). In order to differentiate between structural isomers that eluted at the same retention time, chromatographic deconvolution using the local minimum search algorithm was applied in MZmine. The isomers were separated into individual LC-HRMS features if their chromatographic 3D resolution was sufficient. However, it is a real challenge to get a perfect setting for this function that would work correctly every time. The raw data were manually validated to confirm the output for outlier and bioactive strains only. The adduct and complex search tools were used for the identification of non-proton adducts and complexes, respectively. This minimized mis-assignment of features such as solvent or salt adducts and complexes such as dimers. The formula prediction tool enabled the possible molecular formulae for each feature to be predicted. The elemental composition output was supported by the heuristic isotopic pattern filter in MZmine [47]. The isotope fit scores were calculated for each isotope ion then combined with the individual fit scores which were weighed by their expected intensities. For each ion peak, the *m*/*z* and intensity differences between the expected and the measured patterns were obtained. Those differences were then normalized (normalized deviation values) to the maximum allowed mass and intensity deviation of 0.01%. The relative intensities for the expected and measured values were derived from the isotopic pattern spectra. Each value is a percentage of the isotope’s intensity relative to that of A0. The normalized differences were summed by vector addition of intensity (I) and mass (M) deviations for *m*/*z* ions A0 [X], to A1 [X + 1], A2 [X + 2], and A3 [X + 3] [48].

Positive and negative data were then exported as a CSV file for further clean-up. One limitation of MZmine 2.10 is that data obtained in positive and negative ionization modes cannot be combined; therefore, it is not possible to assign the ionization mode for each feature. Thus, a macro was written in Excel that enabled positive and negative ionization mode data files to be processed together. This enabled the features that were observed in either or both positive and negative modes to be merged for further statistical analysis. Hence, this minimized the risk of missing poorly ionizing compounds only detectable in one mode. For example, phenolic and anthraquinone compounds poorly ionize in positive mode but ionize very well in negative mode [25], therefore such compounds were not deleted from the surveyed peaks.

Another complication when analyzing bacterial extracts is that they are cultured on complex growth media which generates multiple peaks in mass spectrometry and NMR datasets. The culture medium is a complex mixture of constituents and unutilized components that could cause interference in the detection of true bacterial secondary metabolites during dereplication. Therefore, a medium blank was analyzed together with the bacterial extracts in LC-HRMS and NMR experiments during data processing. The obtained features from the blank were regarded as interference and subtracted. A threshold intensity ratio of 1/20 was used if ion peaks (MS) were found in both the medium blank and the sample. The Excel macro was then utilized to extract and remove peaks originating from the culture medium by applying an algorithm to calculate the intensity of each *m*/*z* in both bacterial and medium extracts. This removed features thought to originate from the medium by only keeping those features with peak intensities 20 times greater in the bacterial samples than in the medium. Bacterial extracts were grouped according to their culture medium and this data clean-up step was carried out for each of the six types of culture medium used.

Using the Excel macro, the data were then recombined into CSV files that were utilized for statistical analysis in SIMCA-P V13.0 (Umetrics, Umeå, Sweden) as well as for dendrogram and heat map generation in the R program (version ×64 2.15.2) (R Foundation for Statistical Computing, Vienna, Austria). The Excel macro was also utilized to dereplicate the samples, matching each *m*/*z* found in each bacterial extract with compounds in the AntiMarin database (using a *m*/*z* threshold of + or −3 ppm) to provide details on the putative identities of all metabolites and to calculate the number of remaining unidentified features for each extract. This macro contains a function to identify the top 20 features (ranked by peak intensity) and corresponding putative identities in each sample by creating individual CSV files for each extract. Hits from the database were accessed using ChemBioFinder version 13 (PerkinElmer Informatics, Cambridge, UK) and structures were confirmed by tandem mass spectrometry (MS/MS) and two-dimensional ^1^H-^1^H correlation NMR spectroscopy (^1^H-^1^H COSY). For COSY analysis, spectra from the bacterial extracts were overlaid with spectra from the culture medium to determine signals and cross peaks originating from the culture medium. Three outlier strains were chosen for detailed discussion in this paper to demonstrate the application of different methodologies in dealing with a chemically diverse set of samples showing variation in terms of secondary metabolite production.

### 2.3. Multivariate Analysis for Strain Selection

**Figure 3:**
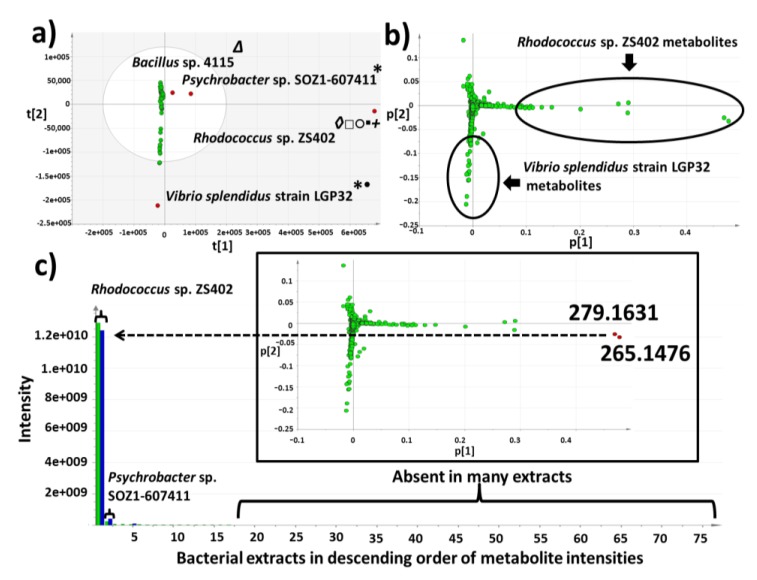
(**a**) Principal component score plot analysis of 77 strains clustered according to features (*m*/*z* ratios) from mass spectral data (*R*_2_ = 0.4)*.* Bioactivities of outliers are represented using symbols; (*) Anti-trypanosomal activity against *Trypanosoma brucei brucei*, (•) PTP1B, (Δ) TRPV1, (◊) TRPA1, (□) TRPM8, (○) PPARα, and (▪) *Enterococcus faecalis*. *Rhodococcus* sp. ZS402 was also found to be NRPS positive (+); (**b**) Accompanying PCA loading plot of the 77 strains investigated in this study; (**c**) Variable intensity plot illustrating two metabolites observed as outliers in (**b**) (*m*/*z* 265.1476 and *m*/*z* in 279.1631) in *Rhodococcus* sp. ZS402.

**Figure 4:**
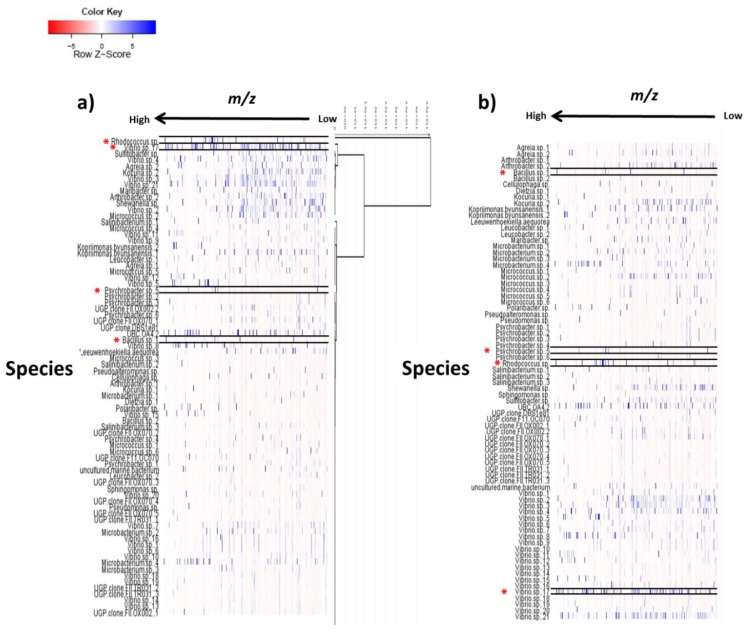
Heat map based on mass spectrometry data displaying distinct metabolic profiles amongst the 77 bacterial species: (**a**) dendrogram from multivariate analysis overlaid with heat map; and (**b**) heat map organized according to species showing differences in the chemical profiles of strains and species. Species observed as outliers from PCA are highlighted and labelled using an asterisk*. (Abbreviations; UBC= uncultured bacterial clone, UGP clone = uncultured gamma proteobacterium, UMB = uncultured marine bacterium).

Processed data was analyzed using SIMCA-P V 13.0 (Umetrics, Umeå, Sweden) using the unsupervised statistical analysis method, principal component analysis (PCA). PCA was used to identify differing features found in the outlying bacterial strains to aid prioritization of the strains with interesting secondary metabolomes. Four predominant outliers, *Bacillus* sp. 4117, *Rhodococcus* sp. ZS402 *Vibrio splendidus* strain LGP32, and *Psychrobacter* sp. were observed, indicating that there was variance in the secondary metabolites produced by these bacterial extracts as they lay furthest from the main group of samples in the score plot (Figure 3a). From the primary general screen, the extracts from these isolates were also found to have interesting bioactivities against *Trypanosoma brucei brucei* and *Enterococcus faecalis*, as well as in target-based functional assays which includes TRPV1, TRPA1 and TRPM8 (pain and cancer) as well as PTPI1 and PPARα (inflammation, diabetes, metabolic disorders and atherosclerosis) (data not shown). *Rhodococcus* sp. ZS402 was also identified as NRPS positive from genetic screening, containing the non-ribosomal peptide genes. The PCA loading plot (Figure 3b) illustrates the features (*m*/*z* ratios, displayed as green dots) that are responsible for the separation shown in the score plot (Figure 3a), indicating the production of unique secondary metabolites particularly by two bacterial strains, *Rhodococcus* sp. ZS402 and *Vibrio splendidus* strain LGP32. Using SIMCA-P it is possible to select any point in the loadings plot to highlight the putative identity of any metabolite and to investigate the peak intensity of this metabolite across the sample set (Figure 3c). Two metabolites (*m*/*z* 265.1476 and 279.1631) were found to be abundant in the *Rhodococcus* sp. compared to the other extracts (Figure 3c); however, they were also observed in the *Psychrobacter* sp. (which was also an outlier in the PCA scoring plot lying adjacent to the *Rhodococcus* sp.) with lower peak intensities, suggesting they both produce some similar metabolites not found in the other extracts. However, further study with *Psychrobacter* sp*.* was halted due to observed instability in the production of the secondary metabolites leading to disappearance of bioactivity after freezing and thawing of the bacterial isolate. It is also worth mentioning that the outlier strains were repeatedly subjected to MS and NMR analysis every three months prior to scale-up work to evaluate their stability. Heat map analysis was utilized to look at the secondary metabolomes in the 77 bacterial extracts (Figure 4a,b) which is a visual representation of the metabolite diversity in the extracts. Multiple blue bands indicate a rich secondary metabolome with a high diversity of metabolites whilst fewer blue bands indicate that a more limited set of secondary metabolites are being produced. Heat maps were overlaid with dendrograms to relate the chemical profiles to the results of the multivariate analysis (Figure 4a). The heat maps were also arranged by species (Figure 4b) to investigate the chemical diversity amongst strains from the same species, exemplified by the 21 strains of *Vibrio* sp. that have very different heat map profiles (Figure 4b). It can be seen that several species from different genera have rich chemical profiles whereas other strains do not (Figure 4a,b). Heat map analysis can also be used to gain an overview of the molecular weight range of metabolites as the features were sorted by *m*/*z* ratios (Figure 4a,b).

### 2.4. Chemical Diversity of Natural Products in Outlying Bacterial Extracts

The limitation of a dereplication study for secondary metabolites, particularly from marine sources, is the difficulty to attain a reference standard for every “hit” from the database. To ensure the correctness of the identification of the basic structure of the identified peaks, UV, MS/MS data and NMR spectral data were used to support the results. However, dereplication through the UV data set was limited only to analyzing chromophore-containing metabolites.

**Figure 5:**
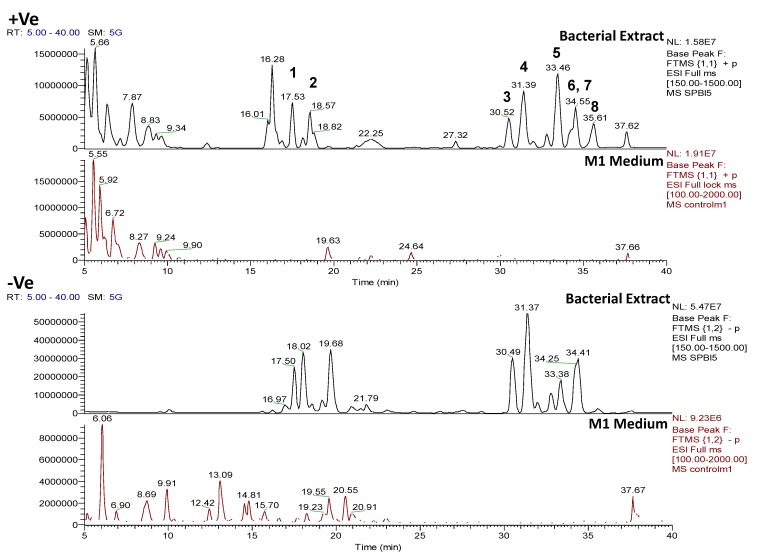
Positive and negative mode base peak chromatograms from outlying bacterial sample, *Bacillus* sp. 4115, annotated to indicate metabolites identified in Table 2. NB: several of the metabolites were detected in both positive and negative modes. Positive and negative mode base peak chromatograms from M1 agar medium are shown to indicate that the annotated metabolites are being produced by the bacteria and are not from the medium.

**Figure 6:**
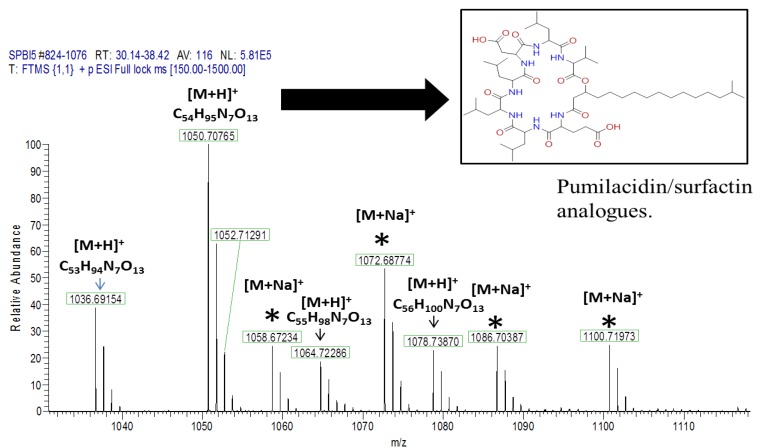
Mass spectrum for *Bacillus* sp. 4115 in the positive ionization mode showing the presence of a cluster of features within the RT range of 30–39 min. Those annotated with an asterisk * are sodium ion adducts, [M+Na]^+^.

**Figure 7:**
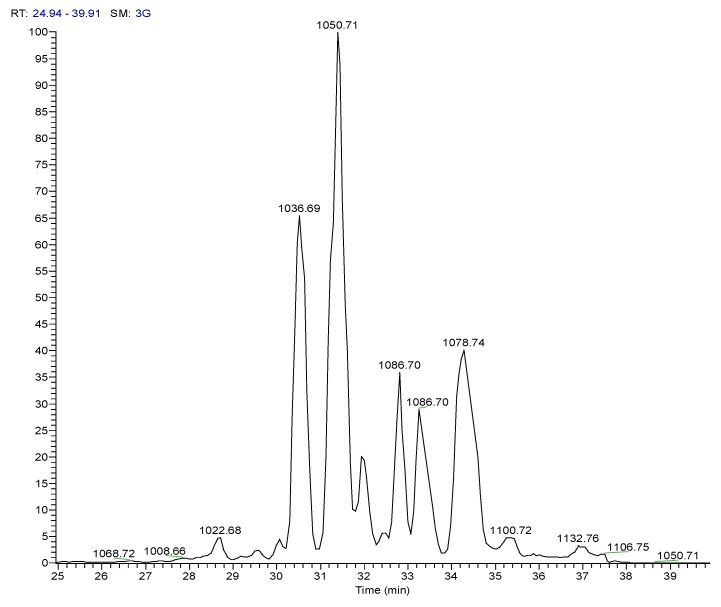
Deconvoluted chromatogram for *Bacillus* sp. 4115 in the positive ionization mode for extracted ions within the *m*/*z* range of 1000–1200 Da.

**Figure 8:**
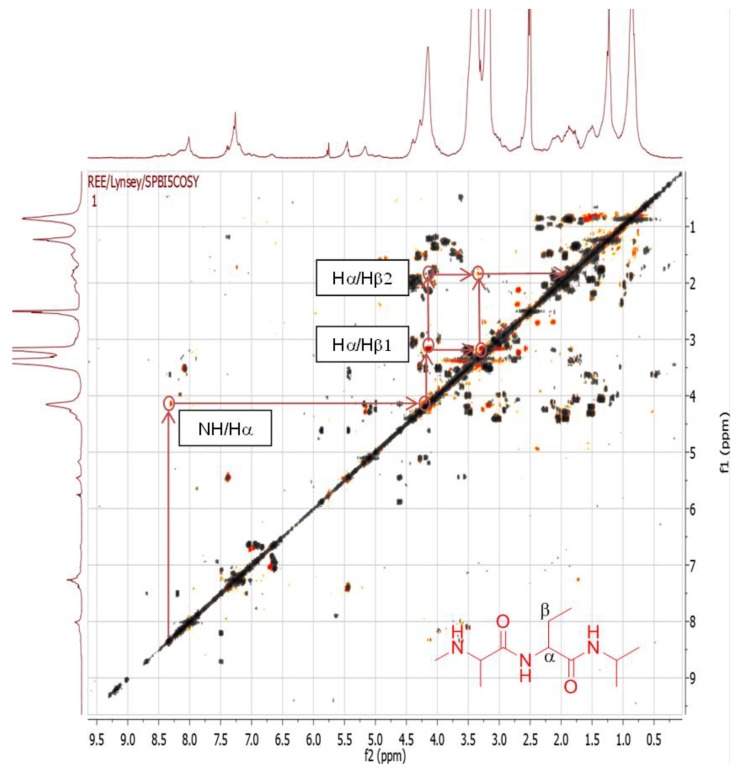
2D-NMR COSY spectrum of *Bacillus* sp. 4115, overlaid with spectrum from M1 medium. Signals in orange are from the sample and signals in grey are from the medium.

**Figure 9:**
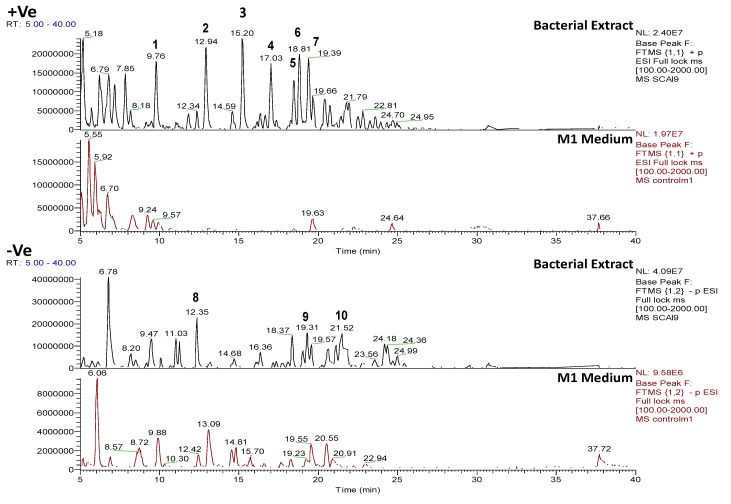
Positive and negative mode base peak chromatograms from outlying bacterial sample, *Vibrio splendidus* strain LGP32 annotated to indicate metabolites identified in Table 3. Positive and negative mode base peak chromatograms from M1 agar medium are shown to indicate that the annotated metabolites are being produced by the bacteria and are not from the medium.

**Figure 10:**
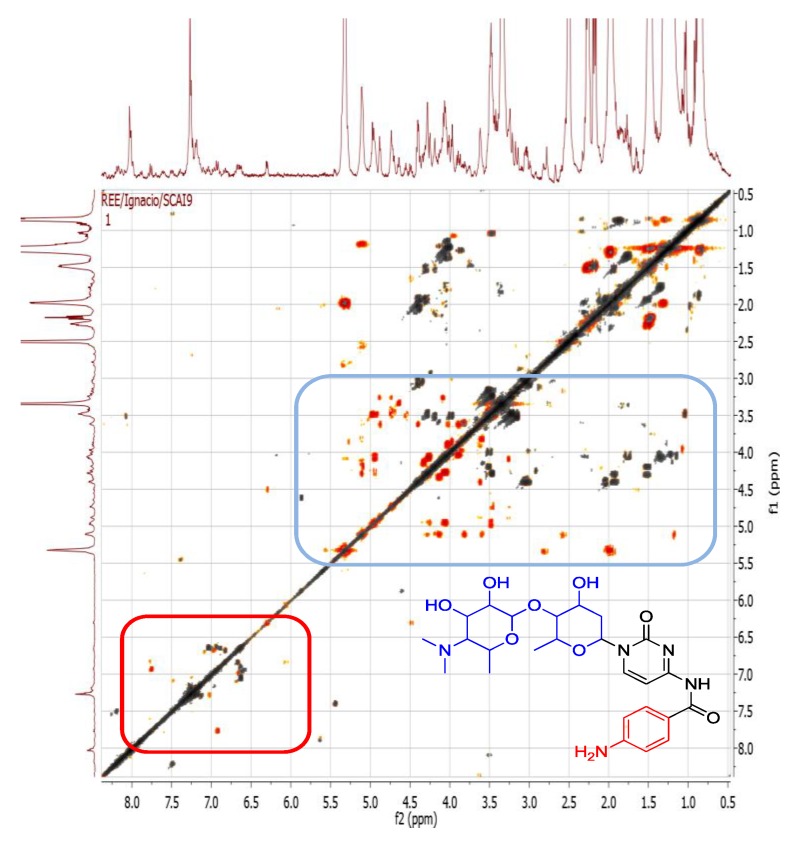
2D-NMR COSY spectrum from sample *Vibrio splendidus* strain LGP32 overlaid with media. Cross signals in orange are from the sample and signals in grey are from the media. Higlighted correlations indicate substructures from oxyplicacetin.

**Figure 11:**
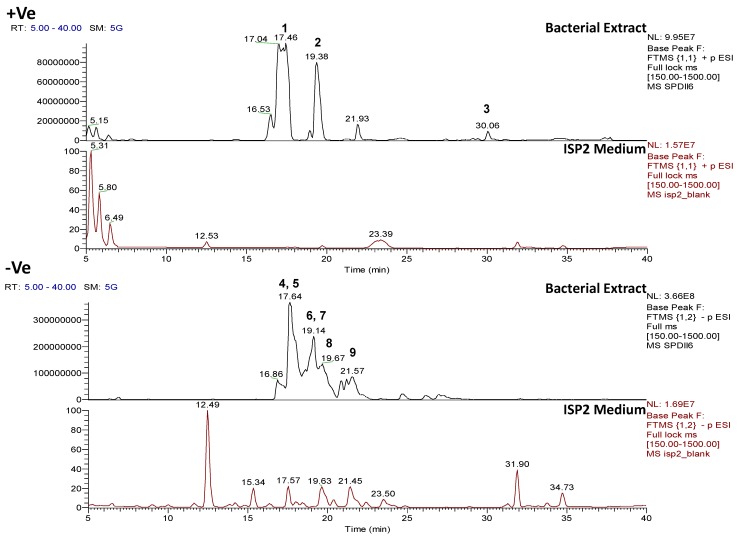
Positive and negative mode base peak chromatograms from outlying bacterial sample, *Rhodococcus* sp. ZS402, annotated to indicate metabolites identified in Table 4. Positive and negative mode base peak chromatograms from ISP2 agar medium are shown to indicate that the annotated metabolites are being produced by the bacteria and are not from the ISP2 agar medium.

**Figure 12:**
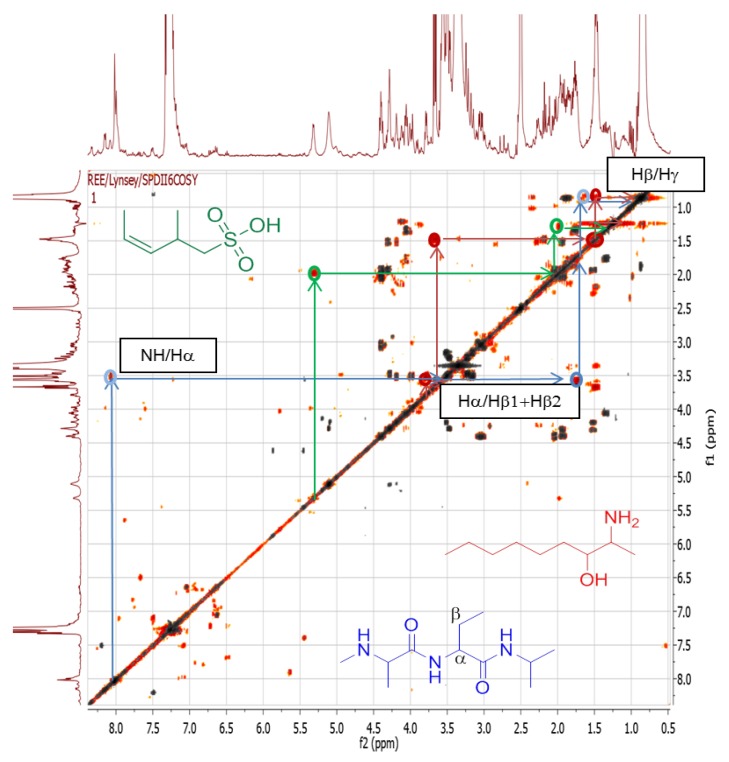
2D-NMR COSY spectrum of *Rhodococcus* sp. ZS402 overlaid with medium. Signals in orange are from the sample and signals in grey are from the medium.

In this study, the mass resolution was 50,000 (at *m*/*z* 400), which is high enough to distinguish isobaric compounds with medium molecular weights (<800 Da). The total number of features identified in three of the outlying bacterial extracts by LC-HRMS is documented in Table 1. The highest number of features was detected in the *Rhodococcus* sp. ZS402, where 1198 features were detected in positive ionization mode and 2361 features were detected in negative ionization mode. By removing features from the ISP2 culture medium, 45% of these features were removed in positive ionization mode, whilst in negative ionization mode 27.4% of these features were removed, leaving 659 in positive mode and 1715 in negative mode. Following dereplication, 28% of features were putatively identified (positive and negative modes combined) whilst 72% were unidentified indicating that this isolate may contain novel compounds (Table 1). The second highest number of features was detected in the *Vibrio splendidus* strain LGP32, where 2767 were detected in positive ionization mode and 654 features were detected in negative ionization mode. By removing features from the M1 medium, 61.2% of these features were removed in positive ionization mode, whereas in negative ionization mode 5.7% of these features were removed, leaving 1102 in positive mode and 617 in negative mode. Following dereplication, 40.7% of features were putatively identified (positive and negative modes combined), whilst 59.3% were unidentified (Table 1). In the *Bacillus* sp. 4115, 1220 features were detected in positive ionization mode and 1037 features were detected in negative ionization mode. By removing features from the M1 medium, 71.6% of these features were removed in positive ionization mode, whilst in negative ionization mode 57.8% of these features were removed, leaving 359 in positive mode and 438 in negative mode. Following dereplication, 51.3% of features were putatively identified (positive and negative modes combined), whilst 48.7% were unidentified (Table 1). Base peak chromatograms and tables listing selected interesting secondary metabolites from three of the outlying bacterial species are shown below (Figure 5, Figure 6, Figure 7, Figure 8, Figure 9, Figure 10, Figure 11 and Figure 12 and Table 1, Table 2, Table 3 and Table 4). The putative identities of metabolites, based on hits from the AntiMarin database, are only given if these metabolites have previously been identified from marine bacteria or sponges.

#### 2.4.1. Dereplication of *Bacillus* sp. 4115

The crude ethyl acetate extract of the *Bacillus* sp. 4115 isolate was active on the initial screen in the target-based functional assay on TRPV1 against pain. Metabolites from the *Bacillus* sp. 4115 extract were putatively assigned as peptides through dereplication (Table 2). They were eluted within the retention time range of 16–38 mins, when the percentage of organic mobile phase (acetonitrile) was greater than 50% (Figure 5, Figure 6 and Figure 7), and could be detected in both positive and negative ionization modes. Several of these were dereplicated using the AntiMarin 2013 natural products database as pumilacidin peptides (surfactins) which have already been described from the marine bacterium *Bacillus pumilus*. Pumilacidins have been described to exhibit antiviral activity [49]. Other plausible congeners that could not be found in AntiMarin were structural analogs of pumilacidins with varying numbers/length of alkyl or peptide side chains that could be targeted for isolation work. It has previously been reported that members of the *Bacillus* genus produce antibiotic peptides as part of their defence mechanism [50]. The fragmentation data reveals the presence of the cyclic and linear moities in the peptides compatible with those of the pumilacidins. The presence of peptides can be further observed by 2D-COSY correlation (Figure 8) of N*H* signals between 8 and 9 ppm with the alpha protons resonating between 3 and 5 ppm which gave additional cross peaks upfield from 1 to 2 ppm, representing the beta proton in amino acid units.

**Table 1:** Summary of the number of features detected in the outlying bacterial extracts: (**a**) total number of features in positive and negative ionization modes (after the removal of features from solvent with intensity >1 × 10^5^); (**b**) total number of features after removal of features from medium; and (**c**) total number of features putatively identified by dereplication (from AntiMarin database) and number of unknowns.

Bacterial Strain	(a) Total number of features (*m*/*z*)		(b) Total number of features (*m*/*z*) after removal of features (*m*/*z*) from medium		(c) Total number of features identified by dereplication with AntiMarin	
	Positive ion mode	Negative ion mode	Positive ion mode	Negative ion mode	Putatively identified (positive and negative modes)	Unidentified (positive and negative modes)
*Bacillus* sp. 4115	1220	1037	359 (29.4% remaining)	438 (42.2% remaining)	270 (51.3%)	526 (48.7%)
*Vibrio splendidus* strain LGP32	2767	654	1102 (39.8% remaining)	617 (94.3% remaining)	699 (40.7%)	1019 (59.3%)
*Rhodococcus* sp. ZS402	1198	2361	659 (55% remaining)	1715 (72.6% remaining)	519 (28%)	1855 (72%)

**Table 2:** Selected metabolites found in *Bacillus* sp. 4115. NB: All of these metabolites were also detected in negative ionization mode.

Peak ID	ESI Mode	*m*/*z*	Rt (min)	Molecular Formula (Isotope Fit Score A0 to A3)	RDB	Hits	Fragmentation Data					
							Fragment ions MS^2^ +Ve	Chemical Formula	RDB	Fragment ions MS^3^ +Ve	Molecular Formula	RDB
1	Pos	445.29092	17.5	C_22_H_40_O_7_N_2_ (99.49%)	4	No hits	427.27921 399.28485 314.19589 232.15408 214.14343 186.14862	C_22_H_39_O_6_N_2_ C_21_H_39_O_5_N_2_ C_16_H_28_O_5_N C_11_H_22_O_4_N C_11_H_20_O_3_N C_10_H_20_O_2_N	5 4 4 2 3 2	168.13794 72.08067	C_10_H_18_ON C_4_H_10_N	3 1
2	Pos	459.30646	18.6	C_23_H_42_O_7_N_2_ (71.70%)	4	No hits	441.29553 413.30063 328.21176 228.15930 200.16431	C_23_H_41_O_6_N_2_ C_22_H_41_O_5_N_2_ C_17_H_30_O_5_N C_12_H_22_O_3_N C_11_H_22_O_2_N	5 4 4 3 2	146.11752 86.09630	C_7_H_16_O_2_N C_5_H_12_N	1 1
3	Pos	1036.69141	30.5	C_53_H_93_O_13_N_7_ (99.82%)	11	Pumilacidin B// (surfactin-1) or other cyclic peptide	1018.67596 937.61896 685.44714 667.43732 455.28571	C_53_H_92_O_12_N_7_ C_48_H_85_O_12_N_6_ C_33_H_61_O_9_N_6_ C_33_H_59_O_8_N_6_ C_22_H_39_O_6_N_4_	12 10 7 8 6	568.36853 342.20117	C_28_H_50_O_7_N_5_ C_16_H_28_O_5_N_3_	7 5
4	Pos	1050.70771	31.4	C_54_H_95_O_13_N_7_ (99.79%)	11	Pumilacidin A// or other cyclic peptide	1032.69104 937.61823 699.46234 681.45282 455.28555	C_54_H_94_O_12_N_7_ C_48_H_85_O_12_N_6_ C_34_H_63_O_9_N_6_ C_34_H_61_O_8_N_6_ C_22_H_39_O_6_N_4_	12 10 7 8 6	568.36816 342.20087	C_28_H_50_O_7_N_5_ C_16_H_28_O_5_N_3_	7 5
5	Pos	875.53519	33.5	C_43_H_77_O_15_N_3_ (98.53%)	7	No hits	710.38348 685.41257 659.46952 654.51534 647.45954 615.44423 610.48905	C_32_H_58_O_15_N_2_ C_31_H_61_O_14_N_2_ C_31_H_67_O_12_N_2_ C_34_H_72_O_10_N C_34_H_65_O_10_N C_29_H_63_O_11_N_2_ C_32_H_68_O_9_N	5 3 1 1 3 1 1			
6	Pos	1078.73917	34.5	C_56_H_99_O_13_N_7_ (99.80%)	11	Pumilacidin C// or other cyclic peptide	1061.72498 966.65216 699.46283 681.45337 455.28549	C_55_H_99_O_13_N_6_ C_49_H_88_O_12_N_7_ C_34_H_63_O_9_N_6_ C_34_H_61_O_8_N_6_ C_22_H_39_O_6_N_4_	10 10 7 8 6	568.36859 342.20135	C_28_H_50_O_7_N_5_C _16_H_28_O_5_N_3_	7 5
7	Pos	889.55163	34.5	C_42_H_76_O_14_N_6_ (84.86%)	8	No hits	861.55371 817.49072 803.47546 790.47894 776.46429 757.47034 690.39093 676.41254 662.39655 590.33954 563.32874	C_41_H_77_O_13_N_6_ C_38_H_69_O_13_N_6_ C_37_H_67_O_13_N_6_ C_37_H_68_O_13_N_5_ C_36_H_66_O_13_N_5_ C_36_H_65_O_11_N_6_ C_31_H_56_O_12_N_5_ C_31_H_58_O_11_N_5_ C_30_H_56_O_11_N_5_ C_26_H_48_O_10_N_5_ C_25_H_47_O_10_N_4_	7 8 8 7 7 8 7 6 6 6 5	449.26096 463.27667 577.34413 477.25619	C_19_H_37_O_8_N_4_ C_20_H_39_O_8_N_4_ C_26_H_49_O_10_N_4_ C_20_H_37_O_9_N_4_	4 4 5 5
8	Pos	903.56635	35.6	C_43_H_78_O_14_N_6_ (99.76%)	8	No hits	817.49097 804.49593 790.47988 718.42297 704.40740 690.42803 676.41285 604.35589 590.34010 577.34508 491.27243 463.27740	C_38_H_69_O_13_N_6_ C_38_H_70_O_13_N_5_ C_37_H_68_O_13_N_5_ C_33_H_60_O_13_N_5_ C_32_H_58_O_12_N_5_ C_32_H_60_O_11_N_5_ C_31_H_58_O_11_N_5_ C_27_H_50_O_10_N_5_ C_26_H_48_O_10_N_5_ C_26_H_49_O_10_N_4_ C_21_H_39_O_9_N_4_ C_20_H_39_O_8_N_4_	8 7 7 7 7 6 6 6 6 5 5 4	364.20825	C_15_H_30_O_7_N_3_	3	

#### 2.4.2. Dereplication of *Vibrio splendidus* Strain LGP32

The ethyl acetate extract of the *Vibrio splendidus* strain LGP32 exhibited biological activity against *Trypanosoma brucei brucei* (marker assay system for trypanosomiasis) and PTP1B. Protein-tyrosine phosphatase 1B (PTP1B) is a novel therapeutic target for type 2 diabetes mellitus, obesity and related states of insulin resistance [51]. *Vibrio splendidus* strain LGP32 contains many semi-polar metabolites indicated by the retention times of the major peaks which ranged from 8 to 25 min (Figure 9). Its LC-HRMS and MS/MS data (Table 3) depicted a highly oxygenated set of metabolites, with the number of oxygen atoms varying from 4 to 13. The ratio of RDBs (ring-plus-double-bond equivalents) to the number of oxygen atoms ranged from 1:2 to 3:5, thus indicating the aromatic nature of the metabolites. COSY correlations between 3 to 5 ppm and 6 to 8 ppm shown in Figure 10 indicate the presence of a glycosidic-like moiety and an aromatic ring system, respectively. The aromatic signals between 6 and 7 ppm signify a phenolic or aniline system. The specified substructures can be found in oxyplicacetin, first detected in the mass spectral dereplication analysis of the isolate (Table 3). Oxyplicacetin, an anti-coccidal agent, was previously isolated from *Streptomyces ramulosus* [52,53].

#### 2.4.3. Dereplication of *Rhodococcus* sp. ZS402

The ethyl acetate extract from *Rhodococcus* sp. ZS402 isolate was found to be active in several of the target-based functional assays, which included TRPA1 and TRPM8 against pain, PPARα in inflammation, diabetes, or other metabolic disorders, as well as against *Enterococcus faecalis*. The chromatographic trace from *Rhodococcus* sp. ZS402, indicated that several interesting features were observed in both positive and negative ionization modes within the retention time range of 16–23 min (Figure 11). Only one of these features was identified using the AntiMarin natural products database during dereplication as xestoaminol C, an unsaturated acyl compound previously described from the sponge *Xestospongia* sp. Several others could not be dereplicated using AntiMarin but were indicated to be structural derivatives with additional C_2_H_4_ on their side chains. Undereplicated features observed in the negative ionization mode specified the presence of sulfated metabolites from the molecular formula identification searches in Xcalibur and MZmine [30], as well as the occurrence of the sulfate fragment ion [HSO_4_]^−^ at *m*/*z* 96.9590 in the MS/MS data (Table 4).

The 2D-COSY spectrum (Figure 12) illustrates that this extract has a rich secondary metabolome. Signals can be seen which correspond to aromatic compounds (6–9 ppm), sugars (4–6 ppm) and sulfated aliphatics (0–4 ppm) and/or olefinics (2–5 ppm). The presence of peptides was also observed by cross peaks exhibited from the N*H* to the alpha and beta proton, typical for an amino acid. Table 5, summarizes the peptide metabolites that were detected in the positive ionization mode. This supports the presence of NRPS genes in the *Rhodococcus* sp. ZS402 bacterium. However, MS/MS data was only achieved for one detected metabolite. In Table 5, the presence of peptides can be determined within a range of double-bond equivalences [47] or alternatively calculated where the RDB is equivalent to [(#_O_ − #_N_)/2 + #_N_] IF linear; (−1) IF linear: (+1) IF cyclic; (+4) for additional Phe/Tyr; (+6) for additional Trp but account for extra Nitrogen(s) when [(#_O_ − #_N_)/2 + #_N_] is less than the found RDB which is also encountered with Arg. Besides following the Nitrogen Rule, approximately every 100 Da represents one amino acid with 1 RDB except for Phe, Tyr, and Trp. However, it was not possible to obtain the fragmentation data for most of the detected ion peaks due to the low intensities of parent ion peaks and/or the conceivable cyclic nature of some of the peptides. Devoid of a chain moiety, in comparison with the pumilacidins found in the *Bacillus* sp. 4115 isolate, cyclic peptides would need a hydrolysis step to cleave the ring prior to further fragmentation.

## 3. Experimental Section

### 3.1. Sample Collection and Bacterial Isolation

Several species of cold water marine invertebrates found in Scottish coastal waters (Orkney Islands, Scotland, UK) were swabbed for microbial symbionts. Six different media were utilized for the isolation of bacteria: M1 [54], ISP2 medium 2 [55], oligotrophic medium (OLIGO) [56], Luria agar (LA), marine agar (MA) [57], and R2A agar [58]. For medium preparation, starch and glucose monohydrate (Alfa Aesar, Heysham, England), yeast extract and malt extract (Oxoid Limited, Hampshire, England), peptone and tryptone (Fisher Scientific, Hemel Hempstead, UK) and glycerol–phosphate, and R2A isolation agar (Sigma Aldrich, Steinheim, Germany) were purchased. All media contained nutrient agar (Oxoid Limited, Hampshire, England) and were prepared using artificial seawater, prepared using Advanced Pro Formula sea salt mix (23 g/L) (Royal Nature, Nesher, Israel).

Plates were incubated at 12 °C which led to the growth of visually diverse colonies of bacteria after 1–4 weeks. Distinct colony morphotypes were picked and bacterial streaking was utilized until pure bacterial colonies were isolated. Bacteria were then maintained on agar plates for short-term storage or archived for long-term storage. To archive the isolates, 3 mL of sterile artificial seawater was added to each plate and mixed before 400 μL of bacteria and artificial seawater solution was transferred into a 2 mL cryovial that contained 400 μL of 30% glycerol (Fisher Scientific, Hemel Hempstead, UK) using a pipette with sterile filter tips.

**Table 3:** Selected metabolites found in positive and negative ionization modes in *Vibrio splendidus* strain LGP32. (P = positive mode; N = negative mode).

Peak ID	ESI Mode	*m*/*z*	Rt (min)	Molecular Formula (Isotope Fit Score A0 to A3)	RDB	Hits	Fragment Ions MS^2^	Molecular Formula	RDB	Fragment ions MS^3^	Molecular Formula	RDB
1	P	219.12266	9.8	C_10_H_18_O_5_ (99.97%)	2	(4*E*)-6,7,9-Trihydroxydec-4-enoic acid	173.08047 133.08556 115.07513 87.04388 73.06467	C_8_H_13_O_4_ C_6_H_13_O_3_ C_6_H_11_O_2_ C_4_H_7_O_2_ C_4_H_9_O	3 1 2 2 1			
2	P	305.1590	12.9	C_14_H_24_O_7_ (99.99%)	3	No hits	259.11685 219.12209 173.08040 155.06985 133.08549 115.07516 87.04391	C_12_H_19_O_6_ C_10_H_19_O_5_ C_8_H_13_O_4_ C_8_H_11_O_3_ C_6_H_13_O_3_ C_6_H_11_O_2_ C_4_H_7_O_2_	4 2 3 4 1 3 2			
3	P	408.22407	15.2	C_18_H_33_O_9_N (Ammonium adduct of C_18_H_31_O_9_) (60.57%)	3	No hits	392.19974 305.15979 259.11786 219.12292	undetermined C_14_H_25_O_7_ C_12_H_19_O_6_ C_10_H_19_O_5_	3 4 2			
3	P	408.22407	15.2	C_19_H_29_O_5_N_5_ (99.50%)	8	No hits	392.19969 305.15978 259.11783 219.12292	undetermined C_15_H_21_O_3_N_4_ C_13_H_15_O_2_N_4_ C_10_H_19_0_5_	8 9 2	173.08086 155.07023 133.08593 115.07541	C_8_H_13_O_4_ C_8_H_11_O_3_ C_6_H_13_0_3_ C_6_H_11_O_2_	3 4 1 2
4	P	494.25967	17.0	C_22_H_39_O_11_N (Ammonium adduct of C_22_H_37_O_11_) (99.97%)	4	No hits	477.23270	C_22_H_37_O_11_	5	459.22238 431.19101 373.18582 345.15396 305.15924 259.11740 219.12263 155.07021	C_22_H_35_O_10_ C_20_H_31_O_10_ C_18_H_29_O_8_ C_16_H_25_O_8_ C_14_H_25_O_7_ C_12_H_19_O_6_ C_10_H_19_O_5_ C_8_H_11_O_3_	6 6 5 5 3 4 2 3
5	P	580.2965	18.5	C_26_H_45_O_13_N (Ammonium adduct of C_26_H_43_O_13_) (99.96%)	5	No hits	563.26880 477.23288 431.19122 345.15424 305.15945	C_26_H_43_O_13_ C_22_H_37_O_11_ C_20_H_31_O_10_ C_16_H_25_O_8_ C_14_H_25_O_7_	6 5 6 5 3	259.11752 219.12265 155.07025	C_12_H_19_O_6_ C_10_H_19_O_5_ C_8_H_11_O_3_	4 2 4
6	P	448.2180	18.8	C_20_H_33_O_10_N (Ammonium adduct of C_20_H_30_O_10_) (91.50%)	5	No hits	431.18991 345.15372 259.11725 241.10663 155.07002	C_20_H_31_O_10_ C_16_H_25_O_8_ C_12_H_19_O_6_ C_12_H_17_O_5_ C_8_H_11_O_3_	6 5 4 5 4			
7	P	534.2550	19.4	C_24_H_39_O_12_N (Ammonium adduct of C_24_H_37_O_12_) (99.13%)	6	No hits	517.22723 431.19070 345.15402 259.11737 241.10681 155.07013	C_24_H_37_O_12_ C_20_H_31_O_10_ C_16_H_25_O_8_ C_12_H_19_O_6_ C_12_H_17_O_5_ C_8_H_11_O	7 6 5 4 5 4			
7	P	534.2550	19.4	C_25_H_35_O_8_N_5_ (71.66%)	11	Oxyplicacetin; 3′-Hydroxy-plicacetin	517.22723 431.19070 345.15402 259.11737 241.10681	C_25_H_33_O_8_N_4_ C_21_H_27_O_6_N_4_ C_17_H_21_O_4_N_4_ C_10_H_17_O_5_N_3_ C_10_H_15_O_4_N_3_	12 11 10 4 5			
8	N	269.13940	12.3	C_14_H_22_O_5_ (99.99%)	5	No hits	251.12892 225.14969	C_14_H_19_O_4_ C_13_H_21_O_3_	6 4			
9	N	405.24944	19.3	C_20_H_38_O_8_ (99.92%)	3	No hits	359.24274 267.19690	C_19_H_35_O_6_ C_16_H_27_O_3_	3 4			
10	N	285.20719	21.5	C_16_H_30_O_4_ (88.36%)	3	Hexadecanedioic acid/ethyl plakortide Z/ethyl didehydro-seco-plakortide Z	267.19641	C_16_H_27_O_3_	4	125.09721 141.12836 185.11803 223.20638	C_8_H_13_O C_9_H_17_O C_10_H_17_O_3_ C_15_H_27_O	3 2 3 3

**Table 4:** Selected metabolites found in *Rhodococcus* sp. ZS402 in positive and negative ionization modes. (P = positive mode; N = negative mode).

Peak ID	ESI Mode	*m*/*z*	Rt (min)	Molecular Formula (Isotope Fit Score A0 to A3)	Hits	RDB	Fragment Ions MS^2^	Molecular Formula	RDB	Fragment Ions MS^3^	Molecular Formula	RDB
1	P	230.2481	17.3	C_14_H_31_ON (99.77%)	Xestoaminol C	1	212.23662	C_14_H_30_N	1			
2	P	258.2793	19.4	C_16_H_35_ON (99.95%)	No hits	1	240.26793	C_16_H_34_N	1			
3	P	597.5208	30.1	C_35_H_68_O_5_N_2_ (99.01%)	No hits	3	337.28409 355.29462 351.29974 369.31042	C_20_H_37_O_2_N_2_ C_20_H_39_O_3_N_2_ C_21_H_39_O_2_N_2_ C_21_H_41_O_3_N_2_	4 3 4 3	319.27350 295.27368	C_20_H_35_ON_2_ C_18_H_35_ON_2_	5 3
4	N	265.1476	17.6	C_12_H_26_O_4_S (99.25%)	No hits	1	96.9590	[HSO_4_]^−^	1			
5	N	760.54162	17.6	C_42_H_75_O_5_N_5_S (90.86%)	No hits	9	531.30280	C_30_H_45_O_5_NS	9	96.9590	[HSO_4_]^−^	
6	N	279.1631	19.1	C_13_H_28_O_4_S (98.04%)	No hits	1	96.9590	[HSO_4_]^−^	1	96.9590		
7	N	816.60400	19.1	C_46_H_83_O_5_N_5_S (87.43%)	No hits	9	279.16318	C_32_H_49_O_5_NS C_13_H_27_O_4_S	9 1	96.9590	[HSO_4_]^−^	
8	N	309.17358	19.7	C_14_H_30_O_5_S (98.04%)	No hits	1	96.9590	[HSO_4_]^−^	1			
9	N	293.1790	21.6	C_14_H_30_O_4_S (99.86%)	No hits	1	96.9590	[HSO_4_]^−^	1			

**Table 5:** Probable peptide metabolites detected in *Rhodococcus* sp. ZS402 isolate in the positive ionization mode. Calculated RDB = [(#_O_ − #_N_)/2 + #_N_] IF linear; (−1) IF linear: (+1) IF cyclic; (+4) for additional Phe/Tyr; (+6) for additional Trp but account for extra N when [(#_O_ − #_N_)/2 + #_N_] < found RDB especially with Arg. Approximately 100 Da represents one amino acid with 1 RDB except for Phe, Tyr, and Trp.

Rt (min)	*m*/*z* [M + H]^+^	Molecular Formula	Isotope Fit Score A0 to A3 (%)	RDB	Predictions to Calculated RDB
19.23	462.1727	C_19_H_23_O_7_N_7_	87.66	12	Cyclic with Phe/Tyr
21.43	499.1871	C_18_H_26_O_9_N_8_	87.64	10	Cyclic
22.15	587.2399	C_22_H_34_O_11_N_8_	82.51	10	Linear
28.14	569.4893	C_33_H_64_O_5_N_2_	99.99	3	Linear
29.46	583.5048	C_34_H_66_O_5_N_2_	99.95	3	Linear
33.77	1078.7151	C_59_H_95_O_11_N_7_	99.86	16	Cyclic with Trp
35.30	1118.7461	C_62_H_99_O_11_N_7_	98.83	17	Cyclic with Trp/Arg
35.64	1092.7308	C_60_H_97_O_11_N_7_ C_55_H_97_O_13_N_9_	99.91 85.21	16 12	Cyclic with Trp Cyclic
36.80	1106.7460	C_61_H_99_O_11_N_7_ C_56_H_99_O_13_N_9_	99.50 99.05	16 12	Cyclic with Trp Cyclic
37.55	849.6953	C_51_H_88_O_4_N_6_ C_56_H_88_O_2_N_4_	99.92 58.43	11 15	Cyclic with Trp/Arg Cyclic with Trp/Arg

### 3.2. Bacterial Culture and Extraction

Seventy-seven fast-growing bacteria were selected from the archive. When required, bacteria in glycerol from archived cryovials were reinoculated onto agar plates and cultured for seven days in a dark incubator at 12 °C. Bacteria were then reinoculated to fresh agar plates by streaking, using disposable sterile loops and cultured as described above for seven days. This step was carried out to get rid of the glycerol in which the bacteria had been stored. Bacteria and agar from three replica plates were then collected into conical flasks using a sterile scalpel to cut the agar into small pieces. Culture growth was terminated with 200 mL HPLC grade ethyl acetate (Sigma Aldrich, Dorset, UK). After 24 h, samples were individually homogenized with an Ultra-turrax T 18 basic homogenizer (IKA, Staufen, Germany), filtered using a Buchner funnel with 110 mm Fisherbrand filters (Fisher Scientific, Hemel Hempstead, UK), transferred to a 500 mL separating funnel and subjected to liquid–liquid extraction and separation. This procedure involved initially separating the aqueous and ethyl acetate phases and washing the aqueous phase twice more with ethyl acetate. Ethyl acetate fractions were then collected, concentrated, weighed and reconstituted for mass spectrometry (1 mg/mL), NMR (5 mg/600 μL solvent) and bioassay screening (10 mg/mL), respectively. The mentioned fixed concentrations were strictly followed for MS and NMR analysis to normalize the weight of biomass used for each of the individual strains.

### 3.3. Mass Spectrometry

Methanol (MeOH), dichloromethane (DCM), acetonitrile (MeCN) and formic acid were purchased (Fisher Scientific, Hemel Hempstead, UK). All reagents were of analytical grade. HPLC grade water was obtained in-house from a direct Q-3 water purification system (Millipore, Watford, UK). Samples and medium control samples were prepared at a concentration of 1 mg/mL in 80:20 MeOH: DCM. A solvent blank was also included. Experiments were carried out using an Exactive mass spectrometer with an electrospray ionization source attached to an Accela 600 HPLC pump with Accela autosampler and UV/Vis detector (Thermo Scientific, Bremen, Germany). The mass accuracy was set to less than 3.0 ppm. The Orbitrap mass analyzer is able to limit the mass error within ±3.0 ppm. The instrument was calibrated to maintain a mass accuracy of ±1.0 ppm by applying the lock mass function. The instrument was externally calibrated according to the manufacturer’s instructions before the run and was internally calibrated during the run using lock masses. In positive ion mode, lock masses were *m*/*z* 83.06037 (acetonitrile dimer) and *m*/*z* 195.08625 (caffeine) and in negative ion mode the lock mass was *m*/*z* 91.00368 (formic acid dimer). Mass spectrometry was carried out over a mass range of 100–2000 *m*/*z* in positive and negative ionization modes with spray voltage of 4.5 kV and capillary temperature at 270 °C. Ten μL was injected from each vial, at a flow rate of 300 μL/min. The column used was an ACE5 C18 column (5 μm × 75 mm × 3 mm) (Hichrom Limited, Reading, UK). A binary gradient method was utilized. The two solvents were A (water and 0.1% formic acid) and B (MeCN and 0.1% formic acid). The gradient was carried out for 45 minutes and the program followed; at zero minutes A = 90% and B = 10%, at 30 min A = 0% and B = 100% at 36 min A = 90% and B = 10% until end at 45 min. The UV absorption wavelength was set at 254 nm, the sample tray temperature was maintained at 4 °C and the column maintained at 20 °C. The samples were run sequentially, with solvent and media blanks analyzed first. LC-MS data was acquired using Xcalibur version 2.2 (Thermo Scientific, Bremen, Germany).

Data-dependent MS^2^ and MS^3^ experiments were carried out using a Finnigan LTQ Orbitrap coupled to a Surveyor Plus HPLC pump (Thermo Scientific, Bremen, Germany) and autosampler (Thermo Fisher, Bremen, Germany) in positive and negative ionization modes using a mass range of *m*/*z* 100–2000 and 30,000 resolution. The capillary temperature was 270 °C, the ion spray voltage was 4.5 kV, the capillary voltage 35 V, the tube lens voltage 110 V and the sheath and auxiliary gas flow rates were 50 and 15, respectively (units not specified by manufacturer). Multi-fragmentation (MS*^n^*) experiments were accomplished on an Orbitrap analyzer, CID (collision-induced dissociation) was utilized with a normalized collision energy of 35%, activation Q of 0.250 ms and activation time of 30,000 ms applied on ions of most intense, 2nd most intense, and 3rd most intense peaks for MS^2^ and MS^3^, respectively, at an isolation width of 3 microns with 5 microscans. Resolution was at 15,000 m/Δm50%, while the minimum ion signal threshold was set to 500. Fragment mass tolerance for molecular formula detection was set at ±5 ppm.

### 3.4. NMR Spectroscopy

Samples were prepared by dissolving 5 mg of bacterial extract (or culture medium extracts as controls) in 600 μL DMSO-d6 (Sigma-Aldrich, Dorset, UK). These were transferred to 5 mm 7″ NMR tubes (Sigma-Aldrich, Dorset, UK). NMR was carried out on a 400 MHz Jeol-LA400 FT-NMR spectrometer system equipped with a 40TH5AT/FG probe (JEOL, Tokyo, Japan). A presaturation sequence was included to suppress the DMSO solvent signal. For presaturation and proton experiments, sixteen scans were recorded while eight scans were recorded for 2D-^1^H-^1^H Correlation Spectroscopy (COSY) analysis. Presaturation and COSY spectra were processed using MestReNova (Mnova 8.1.0) software (Mestrelab Research, Santiago de Compostela, Spain). Normalization, baseline correction with Whittaker Smoother, apodization with Gaussian 1 and smoothing with Savitzy-Golay were carried out in MestReNova. For COSY analysis, spectra from the bacterial extracts were overlaid with the corresponding medium spectrum (control) to differentiate correlations from metabolites produced by the bacteria from those of the culture medium.

### 3.5. Data Analysis Tools for Mass Spectrometry Data

Raw data were initially sliced into two data sets based on the ionization mode (positive and negative modes) using the MassConvert tool from ProteoWizard [59]. The sliced data sets were imported and processed in MZmine 2.10 [30] using predefined settings to extract features from the raw data. The following data processing steps were carried out using MZmine: peak detection, (mass detection and chromatographic builder), deconvolution, deisotoping, filtering, alignment and gap filling. Identification of adducts and complexes and formula prediction steps were carried out to predict possible molecular formulae for each feature and to minimize mis-assignment of features by eliminating adducts and complexes (see Supplementary Information for full details of all settings and procedures utilized to process data in MZmine). Data was then exported as a CSV file for further clean-up.

An algorithm was employed to use the molecular formula data set from Antibase^®^ (February 2013) and Marinlit^®^ (September 2013). These versions are manually curated databases and the given molecular weights do not differentiate between monoisotopic, average, and most abundant masses. The monoisotopic exact masses for each metabolite were then calculated to be used for the customized library. The processed data from MZmine was incorporated into the customized library through the built-in Excel macro for peak identification and dereplication. “Hits” and unidentified peaks were double checked against the MS raw data in Xcalibur 2.2.

Excel macros were written to enable the subtraction of background peaks and to combine positive and negative ionization mode data files generated by MZmine. Peaks originating from the culture medium were extracted. By applying an algorithm to calculate the intensity of each *m*/*z* in both bacterial extracts and medium extracts, ion peaks originating from the medium were subtracted while features with peak intensity 20 times greater in the samples than in the medium were retained. Bacterial extracts were grouped according to their culture media and this data clean-up step was carried out for each culture medium used. The positive and negative ionization mode data sets from each of the respective bacterial extracts were combined by the macro enabling ion peaks that were observed in either or both positive and negative modes to be overlaid for further statistical analysis. The Excel macro was used to dereplicate each *m*/*z* ion peak with compounds in the customized database (using RT and *m*/*z* threshold of ±3 ppm) which provided details on the putative identities of all metabolites in each bacterial extract and sequentially sorted the number of remaining unknowns for each extract. The macro was then utilized to identify the top 20 features (ranked by peak intensity) and corresponding putative identities in each sample by creating a list for each extract. Hits from the database were accessed using ChemBioFinder version 13 (PerkinElmer Informatics, Cambridge, UK). The data was then converted into a CSV file and exported to SIMCA-P V 13.0 Umetrics, Umeå, Sweden), consequentially providing a feature ID number, ionization mode, *m*/*z*, retention time, possible molecular formulae and peak intensity for each feature in all 77 samples. The CSV file was also used to generate a heat map. Heat maps were plotted using the programming software R (version ×64 2.15.2) (R Foundation for Statistical Computing, Vienna, Austria) using a script utilizing the g-plot package. The data set was further analyzed using SIMCA-P V 13.0 using the unsupervised statistical analysis method, principal component analysis (PCA). Dendrograms were also created using SIMCA-P V 13.0 package (Umetrics, Umeå,Sweden).

### 3.6. Molecular Identification

The whole genome DNA of each strain was extracted by scraping bacterial biomass, suspending in 100 μL of sterile water and heating at 95 °C for 10 min before cooling down the lysate on ice and centrifuging at 13,000 rpm for 10 min. The supernatant containing genome DNA was transferred into a new Eppendorf for 16S rRNA gene amplification. For some strains, the genome DNA could not be extracted using the method described above. For these strains, the FastDNA spin kit (MP Biomedicals, Eschwege, Germany) was used to obtain the whole genome DNA according to the manufacturer’s protocol.

Nearly full-length 16S rRNA genes (1542 nucleotide bases) were amplified by polymerase chain reaction (PCR) using primers 27F and 1492R [60]. The reaction mixture consisted of 5 μL of 10× FastDigest green buffer including 20 mM MgCl_2_ (Fermentas, Vilnius, Lithuania ), 1 μL of 10 mM dNTPs mixture (Fermentas, Vilnius, Lithuania), 1 μL of 25 mM of each primer (Sigma, München, Germany), 0.19 μL of 5 U/μL DreamTaq DNA polymerase (Thermo Scientific, Bremen, Germany), 1 μL of template DNA and 41.81 μL sterile water to make a final volume of 50 μL. The PCR was performed on a thermal cycler (Biometra, Goettingen, Germany) using the following thermal cycling protocol: the initial denaturation temperature was 95 °C for 2 min, followed by 34 cyclers of reaction starting another denaturation at 95 °C for 0.5 min, then primer annealing at 56 °C for 0.5 min and primer extension at 72 °C for 1.5 min, as well as the final primer extension at 72 °C for 10 min. The reaction was stopped by chilling at 16 °C to limit the polymerase activity. Five μL of PCR product was examined on agarose gel electrophoresis at 300 V for 20 min. An equal volume of 0.5 μg/μL Genen Rular 1Kb DNA ladder (Fermentas, Vilnius, Lithuania) was used as the reference object. The successfully amplified 16S rRNA genes presenting a clear single band around 1500 bases compared to the ladder under a Molecular Imager^®^ Gel Doc™ XR System (Bio-Rad laboratories, Berkeley, CA, USA) were purified using NucleoSpin Gel and PCR Clean-up package (MACHEREY-NAGEL, Düren, Germany) following the manufacturer’s protocol. The genes amplified with more than one band were purified by cutting off the right band and extracting from the agarose gel using NucleoSpin Gel and PCR Clean-up package according to the manufacturer’s protocol. The concentration of the purified 16S rRNA genes was determined using a NanoDrop 2000C Spectrophotometer (Thermo Scientific, Bremen, Germany) and adjusted to 30 μg/μL. High quality 16S rRNA genes were sent to LGC Genomics GmbH (Berlin, Germany) for initial sequencing using forward primer 27F. Sequences of good quality (usually between 150 and 900 bases) were chosen and contrasted in the GenBank database [61] using the BLASTn tool to identify the nearest neighbour to the amplified sequence. 98% and 95% were used as the thresholds to discriminate between sequences of the same species and genus, respectively.

### 3.7. Bioassay Screening

Extracts were prepared as 10 mg/mL solutions in DMSO in 96-well plate format and delivered for bioassays in dry ice. Extracts were stored at −20 °C until use and then appropriately diluted for testing. Assay-specific thresholds were set to determine the putative active hits: any samples which met this criterion were retested and their activity was assessed over a concentration range of the primary hit. When possible, quantitative measurements of activity (e.g., IC_50_, Ki, MIC) were determined. A full description of assays is presented only for bioactivities observed in extracts from outlier strains.

#### 3.7.1. Anti-Infectives

Bioassays against *Trypanosoma brucei brucei* (model assay system for trypanosomiasis) were carried out as previously described [62]. Bacterial extracts were dissolved in DMSO to prepare 10 mg/mL stock solutions. DMSO was used as the negative control at a concentration of 1% to 0.002% and suramin was used as the positive control at a concentration range of 1 to 0.008 μM. The results were calculated as percentages of control values.

The *in vitro* antimicrobial testing against *Enterococcus faecalis* strain JH212 was carried out using the standard disk diffusion assay [63]. Sterile filter disks were impregnated with the bacterial extracts and placed on agar plates that had been inoculated with the pathogen. After incubation for 24 h, the antimicrobial potential was quantitatively assessed from the diameter of the inhibition zone.

#### 3.7.2. Metabolic Disease and Inflammation

In search for potential drugs against metabolic disorders involving regulation of glucose metabolism, particularly diabetes mellitus and obesity, samples were tested in a protein–tyrosine phosphatase 1B (PTP1B) assay. Samples were tested at 30 μg/mL in duplicate.

#### 3.7.3. Cell-based Functional Assays

Samples were tested at 30 μg/mL in quadruplicate in 384 well plate format. A *Z* factor computation value >0.4 was used to establish primary hits on the initial screen. Cell-based functional assays were carried out on the ion channels involving TRPA1 and TRPV1 (pain), and TRPM8 (pain, cancer) genes, whereas PPARα gene (inflammation, diabetes, metabolic disorders and atherosclerosis) targets a nuclear hormone receptor. The fluorescence readouts for TRPA1, TRPV1 and TRPM8 were measured on a Ca^2+^ sensitive dye as based on Molecular Devices™ [64], while the activity on PPARα was measured against the luminescence on GAL4-UAS luciferase.

## 4. Conclusions

LC-HRMS and multivariate analysis by principal component analysis (PCA) were used to successfully compare the secondary metabolite profiles of crude extracts from 77 respective marine invertebrate-associated bacterial symbionts. PCA was shown to be an effective tool to differentiate bacterial strains based on their chemical diversity and novelty of metabolites, providing a means to select bacterial isolates with diverse chemistry without having to carry out full isolation work on each extract. PCA was used to reveal bacterial species producing similar chemical groups of metabolites grouped together whilst those producing distinct secondary metabolomes were observed as outliers. By using an Exactive mass spectrometer, which enabled fast-polarity switching, it was possible to obtain efficient and greater metabolite coverage in a single experiment, greatly speeding up analysis times. The development of a comprehensive metabolomics workflow pathway including an in-house developed Excel macro embedded with the AntiMarin database made it possible to rapidly dereplicate the 77 strains, providing putative identities of known metabolites in each extract. It was also possible to calculate the number of unknowns in each extract and to produce data files ranking the “top 20 metabolite hits” (ranked by peak intensity) from each strain. This Excel macro also removed peaks associated with the culture medium, making it possible to compare bacterial strains cultured on different types of growth medium and provided data output for statistical analysis. NMR ^1^H and 2D-COSY data was also utilized to confirm the dereplication results obtained from the LC-HRMS data. Additionally, we have shown through PCA and heat map analysis that strains with nearly identical 16S rRNA sequences do not necessarily produce the same secondary metabolites. It is also shown that the dereplication results can also be correlated with bioassay screening results to support drug discovery efforts with the objective of both finding a bacterial isolate that has a unique diverse chemistry and is biologically active. Our approach is to use high resolution MS and NMR in parallel to efficiently detect and confirm the dereplication results. Overall, this shows that metabolomics approaches are worthwhile for the selection of strains for the isolation of novel natural products and that this methodology has the potential to reduce redundancy in drug discovery programs.

## Supplementary Files

Supplementary File 1 Supplementary Information (PDF, 271 KB)
