# Supplementary material for: Metabolomic Tools for Secondary Metabolite Discovery from Marine Microbial Symbionts
Source: Mar Drugs. 2014 Jun 5;12(6):3416–48. doi: 10.3390/md12063416 (PMC4071584; doi:10.3390/md12063416)
Supplement: Supplementary File 1 — Supplementary Information (PDF, 271 KB) [file marinedrugs-12-03416-s001.pdf]

Supplementary Information

Table S1. Results of 16S rRNA gene sequencing indicating closest relative by BLAST search, species, phylogeny, previous known source and cultivation media. Animal sources are annotated follows: Sponges = [1], soft corals = [2], sea urchins = [3].

Media	Sample Code	Source		% Sequence Identity over Sequence Length	Closest Relative by BLAST	Phylogeny
		Animal Source	Previously Reported Source			
ISP2	SPGII2	Hydroid	Subarctic glacial fjord	1310/1310 (100%)	*Salinibacterium* sp. KJF1-8	*Actinobacteria*
ISP2	SPDII6	*Sycon ciliatum* [1]	Antarctic sandy intertidal sediments	1382/1382 (100%)	*Rhodococcus* sp. ZS402	*Actinobacteria*
ISP2	SPCII6	*Grantia compressa* [1]	Subarctic glacial fjord	1422/1431 (99%)	*Psychrobacter* sp. KJF3-38	*Proteobacteria*
ISP2	SPCII1	*Grantia compressa* [1]	Deep sea sediment	1395/1410 (99%)	*Dietzia* sp. O705 K4-1	*Actinobacteria*
ISP2	SPCII4	*Grantia compressa* [1]	Subarctic glacial fjord	1407/1428 (99%)	*Salinibacterium* sp. KJF5-12	*Actinobacteria*
ISP2	SPCII7	*Grantia compressa* [1]	Terrestrial habitat (Sub-Antarctica)	1404/1413 (99%)	*Microbacteriaceae bacterium* MI-5.1P16	*Actinobacteria*
ISP2	SPAII13	*Suberites ficus* [1]	Soil	1394/1401 (99%)	*Micrococcus luteus* strain WS27	*Actinobacteria*
ISP2	SPAII10(A)	*Suberites ficus* [1]	Marine, coastal sediment	1319/1377 (96%)	*Sphingomonas* sp. 2MP11	*Proteobacteria*
LURIA	SPAVII15	*Suberites ficus* [1]	Antarctic soil	1270/1270 (100%)	*Psychrobacter* sp. SOZ1-6074	*Proteobacteria*
LURIA	SPAVII16(B)	*Suberites ficus* [1]	Marine sediments	1329/1329 (100%)	*Kocuria* sp. CNJ770 PL04	*Actinobacteria*
LURIA	SCBVII10(B)	*Mycale (Carmia) similaris* [1]	Silkworm	1334/1334 (100%)	*Bacillus* sp. SW41	*Firmicutes*
LURIA	SCBVII7(A)	*Mycale (Carmia) similaris* [1]	Deep sea sediment	1336/1421 (94%)	*Psychrobacter* sp. es 5	*Proteobacteria*
LURIA	SCBVII7(B)	*Mycale (Carmia) similaris* [1]	Deep sea sediment	1342/1426 (94%)	*Psychrobacter* sp. es 5	*Proteobacteria*
LURIA	SPAVII8	*Suberites ficus* [1]	Jasmine petal	1402/1412 (99%)	*Leucobacter* sp. MLB08	*Actinobacteria*
LURIA	SPAVII9	*Suberites ficus* [1]	Surface water from the Northern Bering Sea	1412/1425 (99%)	*uncult. Gammaproteobacterium* clone DBS1e81	*Proteobacteria*
LURIA	SCBVII11	*Mycale (Carmia) similaris* [1]	*Chionoecetes japonicus* (red tanner crab)	1428/1432 (99%)	*Psychrobacter* sp. CJ-G-PYD3	*Proteobacteria*
LURIA	SPAVII12	*Suberites ficus* [1]	Deep-sea hydrothermal vent sediment	1421/1427 (99%)	*Psychrobacter* sp. YDC2-1	*Proteobacteria*
LURIA	SCBVII9	*Mycale (Carmia) similaris* [1]	Wastewater treatment plant	1415/1417 (99%)	*Pseudomonas* sp. CGMCC 4169	*Proteobacteria*
LURIA	SPAVII7	*Suberites ficus* [1]	Seawater	1377/1386 (99%)	*Micrococcus* sp. S3582	*Actinobacteria*
M1	SPBI5	*Leucosolenia* sp. [1]	Purple paddy soil profile	1087/1097 (99%)	*Bacillus* sp. 4115	*Firmicutes*
M1	SPAI9	*Suberites ficus* [1]	Rainbow trout, west coast of Norway	1255/1255 (100%)	*Vibrio* sp. KV180308-14a	*Proteobacteria*
M1	SPAI11(A)	*Suberites ficus* [1]	*Suberites domuncula*	829/845 (98%)	*Vibrio* sp. 0exn1	*Proteobacteria*
M1	SPAI11(B)	*Suberites ficus* [1]	*Suberites domuncula*	1039/1049 (99%)	*Vibrio splendidus* isolate 28	*Proteobacteria*
M1	SPAiI7	*Suberites ficus* [1]	Rainbow trout, Galacia, Spain	820/836 (98%)	*Vibrio* sp. R117	*Proteobacteria*

Table S1. *Cont.*

Media	Sample Code	Source		% Sequence Identity over Sequence Length	Closest Relative by BLAST	Phylogeny
		Animal Source	Previously Reported Source			
M1	SPCI2	*Grantia compressa* [1]	Mature marine biofilm	789/813 (97%)	*Kopriimonas byunsanensis* strain KOPRI	*Proteobacteria*
M1	SPBI1(A)	*Leucosolenia* sp. [1]	Rainbow trout/coral mucus	877/885 (99%)	*Vibrio* sp. R8	*Proteobacteria*
M1	SPBI1(B)	*Leucosolenia* sp. [1]	Rainbow trout, coral mucus	1300/1313 (99%)	*Vibrio* sp. R8	*Proteobacteria*
M1	SPBI4 (B)	*Leucosolenia* sp. [1]	Coastal marsh	807/831 (97%)	*Microbacterium* sp. OS-6	*Actinobacteria*
M1	SPCI1 (B)	*Grantia compressa* [1]	Rainbow trout	1194/1194 (100%)	*Vibrio* sp. R117	*Proteobacteria*
M1	SPAI8	*Suberites ficus* [1]	*Corylus avellana* L. (Common Hazel).	1234/1234 (100%)	*Microbacterium schleiferi* strain Msc-2	*Actinobacteria*
M1	SPBI3	*Leucosolenia* sp. [1]	Seawater and Fjord water	904/922 (98%)	*Leeuwenhoekiella aequorea*	*Bacteriodetes*
M1	SPBI7	*Leucosolenia* sp. [1]	-	1219/1231 (99%)	*uncultured marine bacterium*	*unknown*
M1	SPEI1	*Diadema* [3]	Arctic Sea Ice	1427/1447 (99%)	*Vibrio* sp. Bsi 20140	*Proteobacteria*
M1	SPCI3(A)	*Grantia compressa* [1]	Heamolymph of spider crab	1431/1434 (99%)	*Vibrio tasmaniensis* strain Mj28	*Proteobacteria*
M1	SPDI4(B)	*Sycon ciliatum* [1]	Macroalgae associated bacteria	1386/1407 (99%)	*Microbacterium* sp. AB320d	*Actinobacteria*
M1	SPDI6	*Sycon ciliatum* [1]	Beach sediment	1440/1445 (99%)	*uncult.* Gammaproteobacterium clone F11-OC070	*Proteobacteria*
M1	SPAI6	*Suberites ficus* [1]	Subarctic glacial fjord	1399/1401 (99%)	*Polaribacter* sp. KJF 12-6	*Bacteriodetes*
M1	SPBI6(A)	*Leucosolenia* sp. [1]	Sea water, Arctic ocean	1332/1377 (96%)	*uncult. bact. clone* OA4-30d-017	*Unknown*
M1	SCBI5	*Mycale (Carmia) similaris* [1]	*Sparisoma* sp. "ninidae"	812/812 (100%)	*Micrococcus* sp. PB7-11B	*Actinobacteria*
M1	SPFI2(B)	*Dead Man's Finger* [2]	Deep sea, *Alphaproteobacteria*	943/943 (100%)	*Sulfitobacter* sp. MBEF09	*Proteobacteria*
M1	SPGI1(B)	*Hydroid*	*Polysiphonia stricta* (red alga)	1401/1415 (99%)	*Shewanella* sp. P1	*Proteobacteria*
M1	SPGI1(D)	*Hydroid*	Haemolymph of the spider crab *Maja brachydactyla*	1416/1424 (99%)	*Vibrio tasmaniensis* strain Mj28	*Proteobacteria*
M1	SCBI4(W)	*Mycale (Carmia) similaris* [1]	Phytoplankton culture in bivalve hatchery	937/937 (100%)	*Vibrio* sp. 2134	*Proteobacteria*
M1	SCBI4(P)	*Mycale (Carmia) similaris* [1]	Antarctic sea sediment	984/984 (100%)	*Kocuria* sp. SS14.13	*Actinobacteria*
M1	SPBI1(C)	*Leucosolenia* sp. [1]	North Sea	788/791 (99%)	*Vibrio* sp. SW5-2	*Proteobacteria*
M1	SPGI1(A)	*Hydroid*	*Chionoecetes japonicus* (red tanner crab)	829/830 (99%)	*Agreia* sp. CJ-G-TSA8	*Actinobacteria*
M1	SPGI1(C)	*Hydroid*	*Chionoecetes japonicus* (red tanner crab)	876/876 (100%)	*Agreia* sp. CJ-G-TSA8	*Actinobacteria*
M1	SPGI3	*Hydroid*	Phytoplankton culture in bivalve hatchery	904/904 (100%)	*Vibrio* sp. 2197	*Proteobacteria*

Table S1. *Cont.*

Media	Sample Code	Source		% Sequence Identity over Sequence Length	Closest Relative by BLAST	Phylogeny
		Animal Source	Previously Reported Source			
M1	SCAI8(A)	*Mycale (Carmia) similaris* [1]	Phytoplankton culture in bivalve hatchery	866/866 (100%)	*Vibrio* sp. 2197	*Proteobacteria*
M1	SCAI9	*Mycale (Carmia) similaris* [1]	*Cassostrea gigas* (Hollow oyster)	760/760 (100%)	*Vibrio splendidus* LGP32	*Proteobacteria*
M1	SPGI5	*Hydroid*	Glacier	858/859 (99%)	*Arthrobacter* sp. TMN-7	*Actinobacteria*
M1	SCBI1(A)	*Mycale (Carmia) similaris* [1]	North Sea	688/688 (100%)	*Vibrio* sp. SW5-2	*Proteobacteria*
M1	SCAI5	*Mycale (Carmia) similaris* [1]	*Delesseria sanguinea* (macroalgae)	939/939 (100%)	*Salinibacterium* sp. AB271d	*Actinobacteria*
M1	SPFI3	*Dead Man's Finger (Soft Coral)*	Marine biofilm	876/880 (99%)	*Kopriimonas byunsanensis* strain KOPRI	*Proteobacteria*
M1	SCAI6	*Mycale (Carmia) similaris* [1]	Soil	850/850 (100%)	*Leucobacter* sp. 4J7B1	*Actinobacteria*
M1	SPGI2	*Hydroid*	Phytoplankton culture in bivalve hatchery	901/901 (100%)	*Vibrio* sp. 2197	*Proteobacteria*
MA	SPCVI8	*Grantia compressa* [1]	*Membranipora membranacea* (Bryozoan)	842/859 (98%)	*Pseudoalteromonas* sp. BB68	*Proteobacteria*
OLIGO	SPGV4(A)	*Hydroid*	North Sea	1274/1286 (99%)	*Cellulophaga* sp. SW5-7	*Bacteriodetes*
OLIGO	SPAiV4	*Suberites ficus* [1]	Aquatic animals "*Latris lineata*"	1430/1434 (99%)	*Vibrio* sp. V004	*Proteobacteria*
OLIGO	SPAiV5	*Suberites ficus* [1]	Oil-polluted subtidal sediments	1387/1389 (99%)	Uncultured *Gammaproteobacterium* clone FII-OX070	*Proteobacteria*
OLIGO	SPAV6(B)	*Suberites ficus* [1]	*Smenospongia* sp.	1395/1398 (99%)	*Vibrio splendidus* strain W221	*Proteobacteria*
OLIGO	SPAV6(C)	*Suberites ficus* [1]	Oil-polluted subtidal sediments	1420/1425 (99%)	Uncultured *Gammaproteobacterium* clone FII-OX070	*Proteobacteria*
OLIGO	SPAV7	*Suberites ficus* [1]	Oil-polluted subtidal sediments	1405/1407 (99%)	Uncultured *Gammaproteobacterium* clone FII-OX002	*Proteobacteria*
OLIGO	SPFeV1	*Dead Man's Finger* [2]	Oil-polluted subtidal sediments	1434/1442 (99%)	Uncultured *Gammaproteobacterium* clone FII-OX070	*Proteobacteria*
OLIGO	SPFeV3	*Dead Man's Finger* [2]	Oil-polluted subtidal sediments	1416/1425 (99%)	Uncultured *Gammaproteobacterium* clone FII-OX002	*Proteobacteria*
OLIGO	SPGV2(A)	*Hydroid*	Sea cucumber "*Apostichopus japonicas*"	1426/1429 (99%)	*Vibrio splendidus* partial 16S rRNA gene, strain ctt 31/5	

Table S1. *Cont.*

Media	Sample Code	Source		% Sequence Identity over Sequence Length	Closest Relative by BLAST	Phylogeny
		**Animal Source**	**Previously Reported Source**			
OLIGO	SPGV2(B)	*Hydroid*	Intestinal microflora of *Haliotis discus hannai*	1427/1442 (99%)	*Vibrio* sp. V004	*Proteobacteria*
OLIGO	SPGV4(B)	*Hydroid*	Oil-polluted subtidal sediments	1417/1418 (99%)	Uncultured *Gammaproteobacterium* clone FII-TR031	*Proteobacteria*
OLIGO	SPC V6(A)	*Grantia compressa* [1]	Deep sea sediment	1404/1406 (99%)	*Arthrobacter* sp. An10	*Actinobacteria*
OLIGO	SPC V6(B)	*Grantia compressa* [1]	Oil-polluted subtidal sediments	1421/1425 (99%)	Uncultured *Gammaproteobacterium* clone FII-OX070	*Proteobacteria*
OLIGO	SPC V6(C)	*Grantia compressa* [1]	Oil-polluted subtidal sediments	1421/1425 (99%)	Uncultured *Gammaproteobacterium* clone FII-OX070	*Proteobacteria*
OLIGO	SPC V7	*Grantia compressa* [1]	Oil-polluted subtidal sediments	1405/1409 (99%)	Uncultured *Gammaproteobacterium* clone FII-TR031	*Proteobacteria*
OLIGO	SPD V6	*Sycon ciliatum* [1]	Oil-polluted subtidal sediments	1417/1428 (99%)	Uncultured *Gammaproteobacterium* clone FII-TR031	*Proteobacteria*
R2A	SCBIII7	*Mycale (Carmia) similaris* [1]	*Sparisoma* sp. "ninidae" (Parrotfish)	1396/1398 (99%)	*Micrococcus* sp. PB7-11B	*Actinobacteria*
R2A	SPAiIII8(A)	*Suberites ficus* [1]	Marine habitat	1410/1416 (99%)	*Maribacter ulvicola* strain KMM 3951	*Bacteriodetes*
R2A	SPAIII6(B)	*Suberites ficus* [1]	*Sparisoma* sp. "ninidae" (Parrotfish)	1384/1385 (99%)	*Micrococcus* sp. PB7-11B	*Actinobacteria*
R2A	SPAIII6 (C)	*Suberites ficus* [1]	*Sparisoma* sp. "ninidae" (Parrotfish)	1384/1385 (99%)	*Micrococcus* sp. PB7-11B	*Actinobacteria*

Settings and procedures utilized to process data in MZmine 2.10

In MZmine, the RAW data is imported by selecting the ProteoWizard-converted positive or negative files in mzML format (Raw data methods → Raw data import). The peaks in the samples and blanks were detected using the chromatogram builder. Mass ion peaks were isolated (Raw Data Methods → Peak detection → Mass detection) with a centroid detector threshold that was greater than the noise level set to 1.0×10^4 and an MS level of 1. Following this, the chromatogram builder (Raw Data Methods → Peak detection → Chromatogram builder) was used with a minimum time span set to 0.2 min, and the minimum height and *m*/*z* tolerance to 1.0×10^4 and 0.001 *m*/*z* or 5.0 ppm, respectively. For all remaining steps, select all files under peak lists before executing each step.

Chromatogram deconvolution was then performed to detect the individual peaks (Peak List Methods → Peak detection → Chromatogram deconvolution). The local minimum search algorithm (chromatographic threshold: 95%, search minimum in RT range: 0.4 min, minimum relative height: 5%, minimum absolute height: 3.0×10^4, minimum ratio of peak top/edge: 3, and peak duration range: 0.2–5 min) was applied. Isotopes were also identified (Peak list methods → Isotopic peaks grouper → Deisotope) using the isotopic peaks grouper (*m*/*z* tolerance: 0.001 *m*/*z* or 5.0 ppm, retention time tolerance: 0.1 absolute (min), maximum charge: 2, and representative isotope: most intense). This step will only deisotope peaks that were detected in the original search *i.e.*, those assigned a peak ID.

Filtering is useful to set certain parameters when only considering a certain RT window e.g., 5–40 min or *m*/*z* range window or to discard IDs that are only present in one sample (Peak List Methods → Filtering → Peak List Rows Filtering). For chromatographic alignment and gap-filling (Peak List Methods → Alignment → Join aligner), the retention time normalizer (*m*/*z* tolerance: 0.001 *m*/*z* or 5.0 ppm, retention time tolerance: 0.5 absolute (min), and minimum standard intensity: 5.0×10^3) was used to reduce inter-batch variation. The peak lists were all aligned using the join aligner parameters set to *m*/*z* tolerance: 0.001 *m*/*z* or 5.0 ppm, weight for *m*/*z*: 20, retention time tolerance: 5.0 relative (%), weight for RT: 20. The values for the weight of *m*/*z* and RT should be kept the same; this means that both RT and *m*/*z* are given equal importance.

Missing peaks (*(peaks undetected by previous algorithms due to deficient peak detection or a mistake in peak list alignments)* were detected using the gap filling peak finder (Peak List Methods → Gap filling: Peak Finder) with an intensity tolerance of 25%, *m*/*z* tolerance of 0.001 *m*/*z* or 5.0 ppm, and retention time tolerance of 0.5 absolute (min). After this step a file will be created called "neg-gap filled" if negative mode and "pos-gap filled" if positive mode. Open the files and after gap-filling delete all peaks found in solvent blanks above a threshold (determined by user).

An adduct search (Peak list methods → Identification → Adduct search) was performed for Na−H, K–H, NH4, formate, and ACN + H (RT tolerance: 0.2 absolute (min), *m*/*z* tolerance: 0.001 *m*/*z* or 5.0 ppm, max relative adduct peak height: 30%). Additionally, a complex search (Peak list methods → Identification → Complex search) was performed (ionization method: $[M + H]^+$ for ESI positive mode and $[M - H]^-$ for ESI negative mode, retention time tolerance: 0.2 absolute (min), *m*/*z* tolerance: 0.001 *m*/*z* or 5.0 ppm, and with maximum complex peak height of 50%). The processed data set was then subjected to molecular formula prediction and peak identification (Peak List Methods → Identification → Formula Prediction) to search for unidentified features. Select atoms C,H,N,O and

any other elements. Adjust parameters with heuristics element count with all three sub-options to get the isotope pattern filter working with all features with isotope peaks.

© 2014 by the authors; licensee MDPI, Basel, Switzerland. This article is an open access article distributed under the terms and conditions of the Creative Commons Attribution license (http://creativecommons.org/licenses/by/3.0/).
